# Pressure and Pain: A Qualitative Pilot Study Describing the Complexity of Unhoused Women’s Experiences with Reproductive Care

**DOI:** 10.1177/26884844251387016

**Published:** 2025-10-08

**Authors:** Casey C. Woods, Anthony L. Shanks, Niki Messmore, Krista J. Longtin

**Affiliations:** ^1^ Indiana University School of Medicine, Indianapolis, Indiana, USA.; ^2^ Hoosier Public Health Corps, Indianapolis, Indiana, USA.

**Keywords:** contraception, homelessness, women’s health, trauma-informed care, shared decision-making, coercion, informed consent

## Abstract

**Introduction:**

While there is extant literature surrounding barriers to reproductive care in the unhoused population—especially regarding access to contraception—little attention has been given to patient perspectives and the quality of care that is received. The unhoused population, which is disproportionately persons of color and low income, has a higher prevalence of psychiatric and physical disabilities. This is a population that has historically been a victim of eugenics and coercive practice.

**Study Objective:**

The purpose of the study is to qualitatively understand the experiences of currently unhoused women with pregnancy, birth control, and sterilization, as well as analyze their perspectives in their clinical encounters over their lifetime.

**Materials and Methods:**

Participants (*n* = 10) were recruited from two shelters in a midwestern city. The sample consisted of those assigned female at birth, are currently experiencing homelessness, and have experience with obstetrical and gynecological health care. A semistructured interview was conducted with each participant utilizing a question bank. Audio was recorded, transcribed, and coded for themes.

**Results:**

The main themes coded from the interviews were negative birthing and/or sterilization experience, lack of shared decision-making for birth control and/or sterilization, pressure to undergo sterilization and/or go on birth control, the desire for a nonjudgmental provider, and sexual violence experience.

**Conclusions/Implications:**

Our data indicate that women experiencing homelessness (WEH) may prefer contraceptive conversations that are trauma-informed and rooted in a shared decision-making model, which in turn may help WEH develop more agency around their reproductive health care. This pilot study also emphasizes the need for more qualitative research to evaluate experiences directly in the population of interest.

## Introduction

In 2023, an estimated 20 per 10,000 people experienced homelessness in the United States, with women accounting for about 4 per 10,000 people.^1^ The unhoused population, which is disproportionately persons of color and low income, has a higher prevalence of psychiatric and physical disabilities, substance use disorders (SUD), as well as heart and lung disease.^1,2^ Persons experiencing housing instability already have higher rates of past trauma than the general population, and being unhoused or housing unstable increases susceptibility to additional traumatic experiences.^3–6^ Women experiencing homelessness (WEH) are more likely to experience an unintended pregnancy, and being pregnant while homeless increases the risk of pregnancy complications, such as hypertension, hemorrhage, and obstetric-related trauma.^3,7^

While access to health care is just one issue for WEH, the ultimate care that is delivered has historically been problematic, as this is a population that has been a victim of eugenics and coercive practices.^8–11^ For example, although increased access to birth control was a step toward female empowerment, its promotion in the early 20th century specifically targeted disabled and poor, Black and Indigenous women in the United States.^8^ The 1990s brought the rise of Norplant, the first long-acting reversible contraceptive (LARC) implant, as it provided a strategy for “dealing with the welfare system, poverty, child abuse and teen pregnancy.”^11^ LARC was presented as a sort of panacea, however, LARC promotion to certain groups was fueled by systemic classism and racism. For example, a 1996 article in the *American Journal of Public Health* instructs family planning counselors on when to use all-options counseling and when to use directive counseling, stating a “substance-abusing [sic] client who is overwhelmed by the circumstances of her life” should simply not have children.^12^ The article goes as far as to say that, if the patient does not want to use a LARC, “the counselor is justified in attempting to use a variety of techniques to change the client’s mind.”^12^ This approach embodies the traditional paternalistic model of medicine—a model which is being phased out and replaced with shared decision-making (SDM).

The SDM model encourages patients to have agency in the diagnostic and therapeutic relationship; more specifically, agency refers to the ability and capability of the patient to contribute, influence, and make decisions within the health care system.^13^ Some barriers to achieving adequate patient agency are uneven power dynamics between patients and clinicians, the persistence of paternalism, and failure to identify that there is a problem at all.^13^ Encouraging patients to have more agency over their health care decisions offers them more control—an opportunity which has frequently been taken away from WEH.

Despite the history of paternalism and the targeting of certain demographics for sterilization or birth control promotion, there is a lack of literature directly including the perspectives of current WEH on their own reproductive health care throughout their lifetime. Extant literature describes barriers to care and suggestions on how to decrease those barriers, especially regarding WEH’s access to contraception.^14–18^ For example, previous research indicates that economic disparities, transportation, shelter conditions, unhealthy relationships, medical literacy, and provider stigma are all barriers to WEH receiving reproductive care.^14,15^ However, little attention has been given to patient perspectives and the quality of care that is received.^14–18^ This article focuses on all aspects of obstetrical and gynecological (ob/gyn) care, not just contraception and pregnancy prevention, but on the lived experience of each participant with the U.S. medical system. Additionally, given the increased stigma around health care for marginalized populations since the COVID-19 pandemic and national restrictions on reproductive care, research understanding the unique experiences of subpopulations is critical to adapting the system to meet patients’ needs.^19^

The objective of our study was to explore the lived experiences of WEH and their reproductive decision-making preferences by conducting qualitative interviews guided by the following research question: What are the perspectives of WEH regarding their reproductive care, specifically pregnancy, birth control, and sterilization?

## Materials and Methods

Our qualitative interview study was approved by our institutional review board. Study participants were recruited from two different shelters in a midsized city in the Midwest, a day shelter and an overnight shelter, both managed by the same nonprofit organization. The organization from which participants were recruited for this study serves approximately 235 women at any given time. Demographic data of the entire population were provided by the organization, and of the 536 individuals served in 2023, approximately 76% are white and 44% are women. Clients of the organization have high rates of trauma; for example, approximately 26% self-report experiencing domestic violence, 44% self-report a mental health disorder, and 28% self-report a SUD.

The interviewer (C.C.W.) was a cisgender white female second-year medical student who is interested in a career in ob/gyn. C.C.W. had volunteered at the shelter for several years prior and was working at the shelter full-time during the summer in which the interviews were conducted. This allowed rapport to be established prior to study onset, which mitigates participant distrust of the researcher and overall research process.^20^ Flyers were posted at the shelters that sought people who have experienced obstetric and gynecological care. Some participants approached the researcher after viewing the flyer, some were approached directly by the interviewer as a convenience sample, and others were recruited via snowball sampling; this method of recruitment was utilized as it is most appropriate for a qualitative study focusing on lived human experiences within a “hard-to-reach” population.^20,21^

Mistrust in scientific research, a fear of harm, stigma, exploitation, or exposure, are barriers to participant recruitment when working with socially disadvantaged populations.^21^ Therefore, no demographic data or names were collected to increase participation and the quality of disclosures. The sample consisted of those who were assigned female at birth, are currently experiencing homelessness, and have had experience with ob/gyn health care; these characteristics were verified through the content of the interviews. C.C.W. conducted the interviews in June through July 2024. Utilizing the qualitative study sample size framework developed by Malterud et al., the sample size of 10 was determined to have adequate “information power” for this pilot study.^22^ The aim of the study is moderately narrow, the population is specific, and the scope of obstetric and gynecological care is defined.^22^ Established theory is applied, and the content of dialogue is strong.^22^

We used a semistructured protocol to guide the interview process. The interview protocol was developed based on the research question and best practices in trauma-informed care (TIC).^23^ We chose to ask broad questions as a strategy to limit retraumatizing experiences for our participants. The unhoused population already has higher rates of past trauma than the general population, and homelessness and precarious housing can lead to environments where additional trauma and abuse can occur.^3–6^ During the interviews, participants were asked about their experiences with reproductive health care, specifically pregnancy, birth control, and sterilization procedures such as tubal ligation and hysterectomy (see Table 1). Participants were given graphics depicting birth control and sterilization methods for education and clarity.

**Table 1.** Example Interview Questions

Have you ever been pregnant? What was your experience like with a doctor, nurse, or other healthcare provider?
Have you ever discussed birth control, such as IUD, the pill, implant, you can refer to the graphic, with a health care provider? If so, can you share how that conversation started and how you felt during that conversation?
Was this conversation before or after your pregnancy? Did you bring up birth control/sterilization or did the doctor? Did the doctor suggest one type over the other?
If you were to go to a doctor or other health care provider to talk about birth control or pregnancy, what would you want to see—what is your ideal experience?

We chose to ask participants for broad narratives describing their experiences of ob/gyn care with unspecified time points (i.e.*,* “What was your experience with contraception like?”), then asked probing questions to clarify the relationship between that event and their current experiences. All participants were experiencing homelessness at the time of their interviews. Although our participants may not have experienced trauma within the ob/gyn environment at the exact same time as being unhoused, our goal was to understand the interplay between housing instability, trauma, and ob/gyn care. The demographics of persons who experience ob/gyn, sexual, and/or violent trauma, psychiatric comorbidities and/or SUDs, and housing instability leading to a state of homelessness are all demographics that overlap with each other and historically have been marginalized.^1,2,8,24^

Interviews took place in an office at the shelter, which was private but not soundproof. All questions outlined in the protocol, along with relevant follow-ups, were asked to each participant. The duration of the interviews ranged from 5 to 50 minutes, depending on the depths of the participants’ answers and narratives. Interviews were recorded, transcribed, and stored on an encrypted device. No field notes were taken during interviews, but clarifying questions were asked. Repeat interviews were not conducted due to the transient nature of the population. The first author cleaned the transcripts and reviewed them for accuracy. Data were stored in Microsoft Excel. C.C.W. and K.J.L. coded the data using Braun and Clarke’s approach to iterative thematic analysis.^25^ Example quotations are chosen for Table 2 that are thought to best exemplify the overall theme.

**Table 2.** Themes and Representative Narratives

Theme	Subtheme	Description	Frequency (*n* = 10)	Example quotation
Negative birthing experience		The experience was negative physically and/or psychologically.	0.70	“They threw my baby against the wall . . . Everybody got hysterical when he was born. The doctor, maybe not the doctor, but the nurses did. I did. [husband] did because [husband] wouldn’t accept the baby and they couldn’t accept that I kept having a [name of other child], like it was like deja vu to them.” -Participant 7
Negative sterilization experience		The experience was negative physically and/or psychologically.	0.40	“Because when they did this…he just had it closed with Steri-Strips instead of stitches, OK. I had metal in my foot; I couldn’t put any weight on the foot because the metal being in it. I had steps to climb up to get into my trailer. My kids’ dad decided I could piggyback. . . The minute he picked me up, this [incision site] opened and gushed, OK? Called the paramedics. They came. They looked at it. He’s like ‘you have another gush like that, there’s going to be a problem.’”-Participant 8
	Medical error	The health care team made a mistake that negatively affected patient’s physical and mental well-being.	0.40	“Yes, with my first pregnancy after I had him. . . they left a thing of gauze up and then sewed it shut, And I don’t know if there was a problem there, but it was horrible I couldn’t walk; people kept calling me ‘lazy mom’ and stuff and I’m like, look there’s something wrong.”-Participant 3
	Inadequate pain control	The patient experienced more pain than they deemed acceptable.	0.40	“I could feel so much pain up here in my stomach, so much that I was about to come out of the restraints. It was that bad and it took them a lot longer than normal.” -Participant 4
	Adverse personal relationships	There was a negative effect on well-being due to relationships with family, friends, partners, etc.	0.40	“They did the second surgery and it was this here surgery. The first surgery, I bounced back…but the second one it kicked my ass and that caused our divorce. You know what he said ‘I’m not changing your bandages.’ He said ‘Your niece moved in to finish high school. Have her do it, it’s gross.’”-Participant 2
Lack of shared decision-making for birth control		There was lack of choice talk/option talk/decision talk during appointment with provider.^20^	0.30	“They don’t tell you the risks. Back then, they didn’t tell you what the risks were. They didn’t tell you should only be on this for 2 years. They say it’s the best thing out there.”-Participant 8
	On Depo-Provera for 2+ years	The patient was on birth control shot for longer than recommended and complained of side effects.	0.20	“And the Depo I would not recommend ever. Never. . . I was with my children’s dad…We were together for three, so I am on it for over 5 years. I stopped taking it because we’re kind of wanting to start a family. Could not get pregnant. . . 6 1/2 years later, I get pregnant. . . so that long with no birth control, I’m not able to get pregnant because of the Depo.”-Participant 9
	Want to be educated on all options	When asked about good qualities in a physician, participant expressed they wanted the provider to explain all family planning options.	0.40	“He told me, how I could get pregnant…And they have, like, an implant they can implant like an egg there to help you get pregnant...So he said if I ever wanted to get pregnant, then to go in there. And I think that was like ideal for me to hear because I didn’t lose hope like in the pregnancy game with PCOS. . . but in my experience I’d rather talk about how I can still have children.”-Participant 1
	Want variety of testing	When asked about good qualities in a physician, participant expressed they wanted the provider to offer a variety of testing.	0.40	“She has micro duplication of chromosome 17. Father, baby’s daddy has micro duplication of chromosome 15, which can cause mental issues…This way the mothers know ahead of time. Hey, this is the problem that you’re going to have with your child.”-Participant 8
Lack of shared decision-making for sterilization		There was lack of choice talk/option talk/decision talk surrounding sterilization.^20^	0.30	Participant 4: “They wanted to take out my uterus for no reason” Interviewer: “So you’re unconscious. So if he [her personal OB/GYN] wouldn’t have came in they would have just taken it all out?” Participant: “Yeah. Yup.” Interviewer: “Did they talk about doing that before?” Participant: “No. it was during the process all together.” Interviewer: “OK, that’s interesting. So no one talked to you before about taking out your uterus, but then they were going to just do it.”
	Had/Expressed need for an advocate	Patient had an advocate or recognizes need for one to improve care and fulfill their wishes.	0.40	“Yeah, he [on call OB/GYN] was there. But at the part where they was going to take it [her uterus] out, they wanted to take it out. Then he [her personal OB/GYN] came in and spoke up and said, ‘hey don’t take that out we can sew it’.”-Participant 4
Pressure to go on birth control		The provider suggested birth control against the patient's wishes.	0.40	“But she wanted to recommend an IUD or something. I didn’t know what that was at the time because I was really young. And I mean, I was in my 20s, 20-something, but I didn’t care for her bedside manner, I guess, because. I just lost my daughter, and she is recommending that I never get pregnant again.”-Participant 1
Pressure to undergo sterilization		The provider suggested sterilization against the patient's wishes.	0.40	“No, I would never want to get my tubes tied. I wish, I’d have as many kids as I could, even if I couldn’t keep them…I think that after my last pregnancy, they kind of pressured me into it.”-Participant 10
Want nonjudgmental provider		When asked about good qualities in a physician, patient expressed desire for a provider who did notjudge them negatively.	0.30	“He was different and very intelligent—caring and nonjudgmental. And he did his job.”-Participant 10
	Judged for SUD	The patient felt judged by their provider due to their history of/current SUD.	0.10	“I want to say that Doctor [name retracted] had the right intention, but I think she judged me based on her medical documents like ‘Ohh well at this rate, this addict will never have a chance of having kids’…I just don’t want to deal with going back to her.”-Participant 1
	Judged for income status	The patient felt judged by their provider due to their low-income status.	0.20	“I think that doctors see you’re low income and think you won’t be able to raise your children. We’ll just keep doing this and we won’t tell you the risks involved and you can find out on your own.”-Participant 8
Sexual violence experience		The participant brought up an experience of sexual violence through the course of the interview.	0.30	“Been raped and so much, so much through my childhood that I had scar tissue on my right fallopian tube…When I got older [it] caused a cyst and scarring over my ovary, so I had to have that removed.” -Participant 10

SUD, substance use disorder; PCOS, polycystic ovarian syndrome; IUD, intrauterine device.

Trustworthiness in the data was established through several methods: (1) ongoing debriefing sessions by the authors to discuss reflections, insights, and interpretations; (2) coding development over 7 months, allowing for comparison with the literature and the recognition of biases and/or distortions; (3) member checking during interviews to elicit the correct interpretation of the narrative, including discussing subthemes. Members of the research team acknowledge their own biases as women in academic medicine while data were interpreted; and (4) member checking with staff at the organization after data were coded to ensure feasibility and reliability. We utilized the Consolidated Criteria for Reporting Qualitative research checklist as a guide for reporting our qualitative research.^26^

## Results

Fourteen individuals were approached, and 10 agreed to participate. No specific rationale was provided for nonparticipants. Eight themes and nine subthemes were coded from interview audio recordings and transcripts. The themes present in our sample are negative birthing, sterilization, or birth control experience; lack of SDM for birth control or sterilization; pressure to go on birth control or undergo sterilization; desire for a nonjudgmental provider; and sexual violence experience. Subthemes were created if more than one person had a similar experience within the overarching theme; for example, the subtheme “medical error” contributed to the overall theme of “negative birthing experience.” The subthemes are displayed in Table 2 in the row below their overarching theme.

Seven of the ten participants detailed narratives about a negative birthing or sterilization experience, with the experience being negative physically and/or psychologically. Participant 7 discussed the birth of her second child with a congenital disability, in which her child was “threw against the wall” by her “hysterical” husband, because he was upset about having another child with a disability. During a sterilization procedure, participant 4 said they felt “so much pain up here in my stomach, so much that I was about to come out of the restraints,” the restraints that were placed due to the patient moving around due to said pain.

Another theme extrapolated from the interviews was frustration over a lack of SDM surrounding birth control and sterilization. Participant 8, reflecting on her experience with birth control, said “They don’t tell you the risks. Back then. They say it’s the best thing out there.” She didn’t feel as through her provider adequately explained the risks or side effects of the birth control she was prescribed.

Many interviewed participants revealed that they felt pressured to go on birth control or be sterilized, with one participant saying “No, I would never want to get my tubes tied…I think that after my last pregnancy, they kind of pressured me into it” (Participant 10). Another participant, after the loss of her child during delivery, was recommended an intrauterine device (IUD) by her doctor and said, “I had just lost my daughter, and she is recommending that I never get pregnant again” (Participant 1).

Participants expressed their frustration over judgments they felt their physician was making. One noted, “I think she judged me based on her medical documents like, ‘Oh well at this rate, this addict will never have a chance of having kids’” (Participant 1). Another woman detailed her frustration about the assumptions made about her life saying, “I think that doctors see you’re low-income and think you won’t be able to raise your children. We’ll just keep doing this and we won’t tell you the risks involved, and you can find out on your own” (Participant 8). One participant was having a cesarean section to deliver her child, and the ob/gyn on shift wanted to take out her uterus, indicating it was too damaged to repair, although the patient indicated they were not aware of the possibility for a hysterectomy prior (Participant 4). The patient’s personal ob/gyn arrived in the operating room and said, “hey don’t take that out; we can sew it,” so the patient did not undergo a hysterectomy (Participant 4). The participant said, “they wanted to take out my uterus for no reason” and was very upset about the initial physician’s proposed course of action, and grateful that another doctor advocated against her sterilization (Participant 4).

## Discussion

Our study demonstrates that WEH report a loss of agency in clinical encounters and that there is a need for a framework that minimizes the effect of clinician and systemic biases. This framework would ideally increase patient satisfaction and trust in their provider. These findings align with recent research on unhoused women’s interconception care (ob/gyn care in between pregnancies) that reveals the need for autonomy while acknowledging the role personal relationships and unstable housing play in WEH’s health care.^27^ The extrapolated themes and the language in participant’s examples indicated to us that there was a need to approach this population specifically with an SDM and TIC framework.

Given the participants’ experiences with paternalism and marginalization in the health care system, we employed the theoretical framework of SDM to illustrate how agency could change the relationship between these patients and their provider(s). As described previously, SDM is a physician-patient communication model in which the patient plays an active role in their care. This is in contrast to the traditional paternalistic model, which places most of the power in the hands of the physician. Using the SDM model developed by Elwyn et al., SDM is composed of three phases: choice talk, option talk, and decision talk.^28^ Choice talk involves identifying the problem and informing the patient that they will have a choice in their care and that their own values and preferences will play a role in that decision.^28^ Option talk involves informing the patient of all their options, even the ones the clinician does not prefer, while decision talk entails the clinician walking through the patient’s preferences and, of course, adding their own expertise.^28^ The clinician must ensure the patient is ready to decide and is adequately informed of their options before beginning the patient’s care plan. If the patient chooses to have the physician make their decision for them, as it is their right to do, then the patient has at least been informed about the options, benefits, and risks that the physician is considering.

Utilizing the SDM model, a clinician would inform the patient of all options, even the ones the clinician thought to be inferior or unrealistic. For example, an unhoused patient (Participant 1) with polycystic ovarian syndrome (PCOS) was informed about in vitro fertilization (IVF) as a means to achieve pregnancy by her ob/gyn. She said, “I’d rather talk about how I can still have children” (Participant 1) as opposed to conversations about birth control and how *not* to have children. Although IVF is quite costly and time-consuming in the United States, and this patient was currently unhoused and struggling financially, the patient still wanted to know that there is a way to have children, even if it was unattainable now. This interaction shows that the physician valued her autonomy and presented the same options as they would to a woman who *did* have the social support and financial means.

Ideally, the SDM model would prevent the patient from feeling pressured into making a specific decision. Several women in the study expressed that they felt pressured by their provider. For example, after the loss of her child during delivery, a study participant was recommended an IUD by her doctor and said, “I had just lost my daughter, and she is recommending that I never get pregnant again” (Participant 1). In other specialties, aspects of paternalism are more acceptable, for example, choosing what blood pressure medication to prescribe, but in obstetrics and gynecology, paternalism can result in the physician deciding who gets to bear children and when. In this setting, especially when working with WEH, extra care could be used to ensure an SDM model is in place, thus empowering patients to make decisions based on their lived experiences.

In addition to SDM, our data indicate that WEH would prefer a TIC model in the reproductive care setting. The main tenets of TIC are trauma awareness, an emphasis on safety, granting opportunities to rebuild control, and a strengths-based as opposed to a deficits-based approach.^23^ This approach involves understanding that the way the patient thinks and behaves can be a result of trauma.^23^ Homelessness in and of itself is a traumatic experience, and our participants had additionally experienced sexual trauma and trauma specific to obstetrics and gynecology. Throughout the course of the interviews, sexual assault was brought up unprompted by 3 of the 10 participants while detailing their experience with reproductive care.

In addition to these disclosures, some of the interviewed women revealed certain birthing or sterilization experiences that were overwhelmingly negative. For example, after childbirth, a piece of gauze was left in Participant 3′s body, which subsequently became infected and increasingly painful; she stated, “It was horrible, and I couldn’t walk. People kept calling me ‘lazy mom’ and stuff, and I’m like, look, there’s something wrong” (Participant 3). Another participant detailed a narrative in which she was using crutches after a combined orthopedic operation and hysterectomy; upon stepping up into her trailer home, the incision site closed with Steri-Strips ripped open, causing her to fall and lose what the paramedics deemed an alarming amount of blood (Participant 8). Patients who have had experiences such as these or who have felt judged or pressured by their physician in the past expressed a need to feel safe with and respected by their provider. In April 2021, the American College of Obstetricians and Gynecologists (ACOG) recommended the implementation of TIC for all patients, especially recognizing the tendency for certain populations to be revictimized by medical care.^29^ Physical and psychological safety are of utmost importance with working with WEH, and a TIC approach can help the physician achieve this in their exam room.^29^ Based on our data, participants’ care was not informed by these approaches.

Previous literature has indicated that SDM and TIC models benefit patients broadly and specifically improve outcomes for patients traditionally underserved in the health care system.^19^ Our data affirm these outcomes, and professional organizations are moving in this direction. For example, in February 2022, ACOG recommended SDM and patient-centered contraceptive counseling for underserved women that recognize historical mistreatment and provider biases and focuses on the priorities and lived experiences of the patient.^30^ In order to implement ACOG’s recommendations, studies such as this one are needed to better understand the preferences and complex experiences of women facing homelessness.

Furthermore, SDM and TIC models can be implemented at the individual and system-wide level.^31,32^ At the individual level, providers should be aware that, specifically when treating WEH, they may make assumptions about a patients’ care preferences and therefore not engage in appropriate choice, option, and decision talk.^28^ Utilizing a graphic for birth control and sterilization counseling would help standardize the process and ensure no options are omitted. Additionally, the patient must understand the options, not just see or hear them, so patient education at the level the patient can comprehend is important. On the topic of family planning, based on our participants’ experiences, it is important to discuss not only contraception but to mention how pregnancy could be achieved when working with patients experiencing homelessness or who are members of other vulnerable groups; this ensures that they do not leave the encounter feeling like their doctor doesn’t think they should ever reproduce. A TIC approach, for providers, involves ensuring the comfort and privacy of patients, explaining sensitive exams, asking for consent, and a general understanding that patients’ reactions and hesitations could be a product of past trauma.

At the system-wide level, more research is needed to understand the impact of SDM and TIC training. Our participants indicated an awareness of clinical time constraints and varying levels of health literacy. To increase the quality of SDM, patients could be offered educational tools and/or decision aids prior to the appointment to allow more time for comprehension and contemplation. Follow-up calls could be scheduled for patients who do not want to decide that day in the office, in order to avoid the pressure of the physician or time restraint. Clear TIC protocols for sensitive exams system-wide would increase the comfort of all patients and avoid retraumatization for others. Recognizing that WEH has an overall higher level of trauma, inside and outside of the exam or operating room, is key to equitable care.^3,5,23^

## Limitations

This study was conducted at two shelters managed by the same nonprofit in a midwestern city. Specific demographic data were not collected to encourage participation; while we know this decreases the generalizability of the data, it was an important step to increase participants’ comfort giving interviews involving sensitive topics, including but not limited to drug use, violence, and medical trauma. We concede that many of the experiences described by our participants occurred at various times in their lives, when their housing may or may not have been unstable. Given our goal of describing and understanding patients’ experience of the interplay between housing insecurity and other trauma, we believe this approach is appropriate for this level of inquiry. We seek to illuminate the complexity of these patients’ experiences and their need for specialized care, rather than claiming a cause/effect or correlative relationship. Though our pilot study only contains 10 participants, it was determined to have enough “information power” based on the framework of Malterud et al.^22^ Another study with a larger sample size and the same cross-case analysis would provide greater insight into the experiences of the population. The interviewer was a cisgender white female second-year medical student who is interested in a career in ob/gyn and had volunteered with the organization for 4 years, working full-time at the time the interviews were conducted. Due to this prior relationship with participants, the results may be different than if the interviews were conducted by an outsider. The interviewer has no personal experience with homelessness or a personal adverse ob/gyn experience. Convenience sampling introduces sampling bias, and the participants who chose to participate may not represent the broader population of WEH. While snowball sampling is beneficial for recruitment, bias can be introduced, and the resulting group can be too homogenous, limiting the diversity of perspectives.

## Conclusions

Our data indicate that WEH may prefer contraceptive conversations that are trauma-informed and rooted in a SDM model, which in turn may help WEH develop more agency around their reproductive health care and mitigate clinician and systemic biases. Patient autonomy, education, and the avoidance of retraumatization are at the core of the proposed clinical model. Additional qualitative research is needed to understand the lived experiences of marginalized demographics directly from and within the population of interest.

## Supplementary Material

Supplementary Data

## Abbreviations Used

ACOG: American College of Obstetricians & Gynecologists
IVF: in vitro fertilization
LARC: long-acting reversible contraceptive
PCOS: polycystic ovarian syndrome
SDM: shared decision-making
SUD: substance use disorder
TIC: trauma-informed care
WEH: women experiencing homelessness
